# Heat sensitivity of first host and cercariae may restrict parasite transmission in a warming sea

**DOI:** 10.1038/s41598-022-05139-5

**Published:** 2022-01-21

**Authors:** Dakeishla M. Díaz-Morales, Claudia Bommarito, Jahangir Vajedsamiei, Daniel S. Grabner, Gil Rilov, Martin Wahl, Bernd Sures

**Affiliations:** 1 Aquatic Ecology and Centre for Water and Environmental Research, University of Duisburg-Essen, Essen, Germany; 2 Benthic and Experimental Ecology Department, GEOMAR, Helmholtz Centre for Ocean Research, Kiel, Germany; 3 Israel Oceanographic and Limnological Research, National Institute of Oceanography, P.O. Box 8030, 31080 Haifa, Israel; 4 Marine Biology Department, The Leon H. Charney School of Marine Sciences, University of Haifa, Mt. Carmel, 31905 Haifa, Israel

**Keywords:** Ecology, Zoology, Systems biology, Nonlinear dynamics, Time series, Parasitology, Parasite biology, Parasite host response, Climate-change ecology, Ecological epidemiology

## Abstract

To predict global warming impacts on parasitism, we should describe the thermal tolerance of all players in host–parasite systems. Complex life-cycle parasites such as trematodes are of particular interest since they can drive complex ecological changes. This study evaluates the net response to temperature of the infective larval stage of *Himasthla elongata*, a parasite inhabiting the southwestern Baltic Sea. The thermal sensitivity of (i) the infected and uninfected first intermediate host (*Littorina littorea*) and (ii) the cercarial emergence, survival, self-propelling, encystment, and infection capacity to the second intermediate host (*Mytilus edulis* sensu lato) were examined. We found that infection by the trematode rendered the gastropod more susceptible to elevated temperatures representing warm summer events in the region. At 22 °C, cercarial emergence and infectivity were at their optimum while cercarial survival was shortened, narrowing the time window for successful mussel infection. Faster out-of-host encystment occurred at increasing temperatures. After correcting the cercarial emergence and infectivity for the temperature-specific gastropod survival, we found that warming induces net adverse effects on the trematode transmission to the bivalve host. The findings suggest that gastropod and cercariae mortality, as a tradeoff for the emergence and infectivity, will hamper the possibility for trematodes to flourish in a warming ocean.

## Introduction

Climate change-related temperature shifts are recognized as one of the main drivers of marine benthic community changes^1–3^. However, in realistic natural scenarios, climate change impacts should be addressed as a concert of multiple abiotic and biotic factors^4–6^. Biotic factors such as species interactions can have the capacity of buffering or amplifying climate change effects on individual species as well as on communities (e.g. Ref.^7^). Thus, to more realistically predict climate change impacts on species or communities, we need to describe the respective thermal tolerance of closely interacting species systems. Host–parasite systems are one example of such interacting systems that, in response to temperature, can result in complex ecological changes^8–12^. Moreover, as many free-living species are infected with at least one specific parasite species^13,14^, host–parasite interactions are among the most intimately interspecific interactions in ecology. Thus, parasites have to be considered in studies of climate change effects as an important group of biotic drivers. In other words, understanding the fate of host–parasite systems in the context of global warming is crucial and demands consideration of the thermal sensitivity of various life-cycle stages of the involved species.

Trematode parasites are of particular interest in the context of global warming due to their complex life cycle, which often includes three hosts: a first intermediate host (usually a gastropod), a second intermediate host (e.g., crustaceans, fish, bivalves, amphibians), and a final host (often a vertebrate such as shorebirds^15^). Such a complex life cycle poses a severe constraint to trematode populations faced with global warming due to two main reasons. First, the absence of a single required host group will directly result in the excision of the parasite from the community in question. Second, the life cycle includes free-living larval stages (i.e., miracidia and cercariae) directly, and potentially differentially, influenced by multiple abiotic and biotic factors from the external environment^16,17^.

Cercariae have been long known to be sensitive to temperature and one of the most fragile components of the trematode life cycle^17,18^. Their survival and self-propelling capacity, for example, are highly constrained by warm temperatures due to the lack of feeding ability and, thus, rapid depletion of energy reserves at warmer temperatures^18^. However, an increase in temperature can benefit other traits such as cercarial emergence and infectivity^19,20^. The observations on this thermally-induced tradeoff in favor of higher emergence and infectivity previously suggested that trematode infections will increase in a warming environment^9,19,20^. However, growing evidence has shown that the matter is more complicated than previously envisioned, mainly due to important factors such as the hosts’ sensitivity to abiotic stressors, the phenology timeline of both hosts and parasites, and the parasites’ species-specific idiosyncrasy^6,10,21–28^.

Considering the first intermediate host's performance in response to temperature is essential to understand the fate of trematodes under global warming. Trematodes show a high specificity for their first intermediate host^15,29^. This high specificity results in reduced flexibility when searching for appropriate hosts. Even though first intermediate hosts (e.g., gastropods) inhabiting intertidal zones are evolutionarily adapted to stressful environments, even these eurytherms may already live close to their limits of thermal tolerance, and prolonged exposure to heat and recurrent heatwaves can push them over the edge^30^. The heat sensitivity of first intermediate hosts can be amplified when they are infected by trematode parasites, especially by castrating parasite species, which can take a considerable toll on its host's thermal sensitivity^31^. Hence, the parasites’ greater production of transmission stages could be offset by increased mortality of the first host in warming oceans.

**Figure 1:**
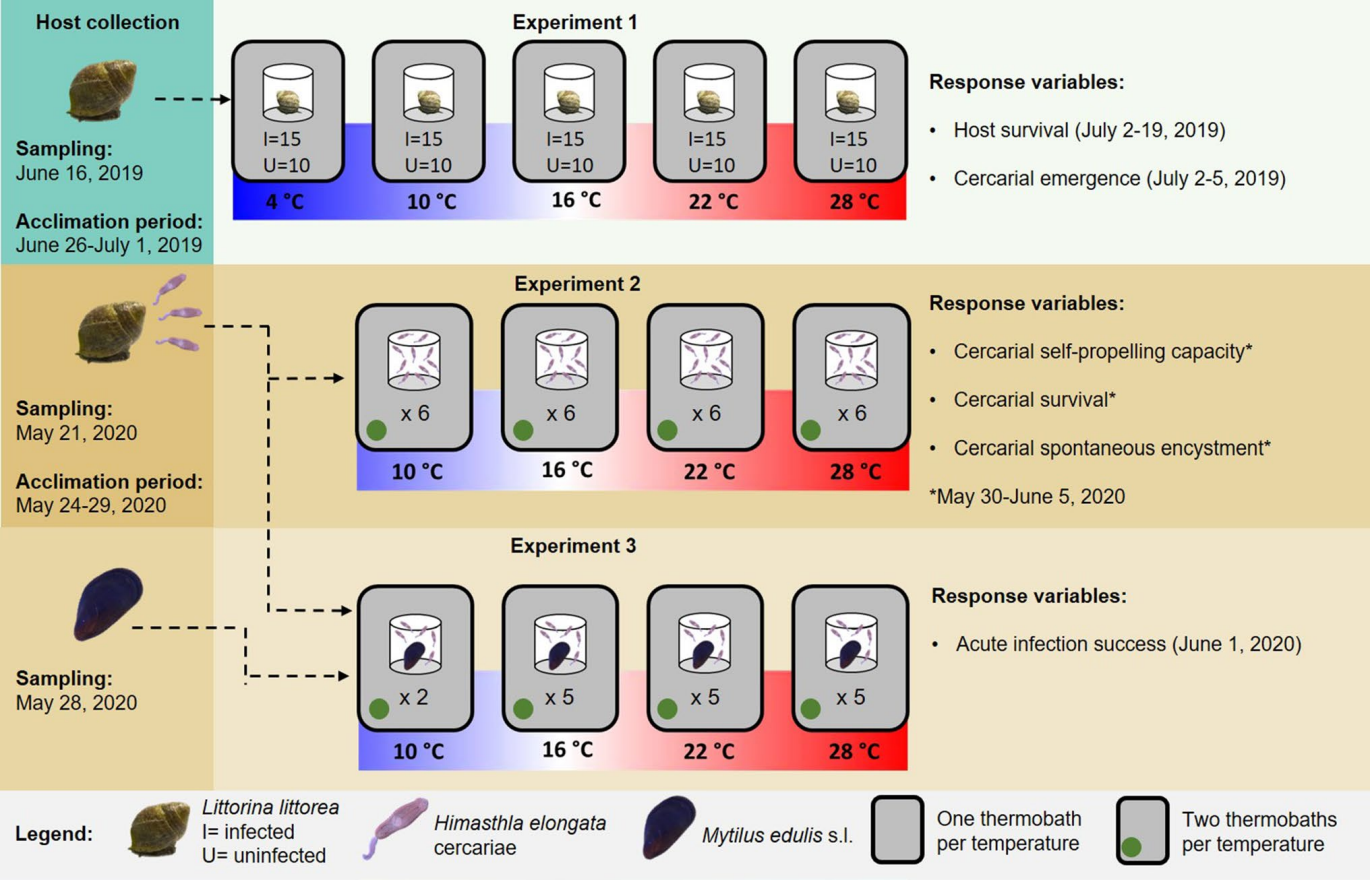
Schematic representation of the experimental design. In Experiment 1, the survival of infected and uninfected *Littorina littorea* was evaluated along with cercarial emergence at 4, 10, 16, 22, and 28 °C. In Experiment 2, the performance of cercariae at 10, 16, 22, and 28 °C was assessed by evaluating their self-propelling capacity and survival and encystment rates. In Experiment 3, acute infection success of *Himasthla elongata* to *Mytilus edulis* s.l. was evaluated at 10, 16, 22, and 28 °C.

Therefore, to understand the net effect of global warming on host–parasite interactions, it is necessary to consider the most fragile components of the life cycle of trematodes, such as the cercariae and the first-intermediate host. Using *Himasthla elongata* (Mehlis, 1831) and its intermediate hosts from the Western Baltic Sea as a model system, this study experimentally assessed the impacts of temperature on (i) the survival of infected versus uninfected first intermediate host (i.e., the snail *Littorina littorea* (Linnaeus, 1758)) and (ii) the emergence of *H. elongata* cercariae along with their self-propelling capacity, survival, encystment, and infectivity to the second intermediate host (i.e., the Baltic *Mytilus edulis* sensu lato (Linnaeus, 1758); Fig. 1). The temperatures tested (4, 10, 16, 22, and 28 °C) represent the range of temperatures expected to be relevant to the performance of the parasite^20^, and include projected end-of-century summer thermal averages and current summer heatwave events in the summer in the Baltic Sea (22 °C)^32–34^, as well as an extreme temperature scenario (28 °C). Specifically, we tested whether the vulnerability of the cercariae and (infected) gastropod host to high temperatures can set a limit on the heat-induced proliferation of the trematode infection to mussels.

## Results

### Experiment 1a: General host infection status and survival

**Figure 2:**
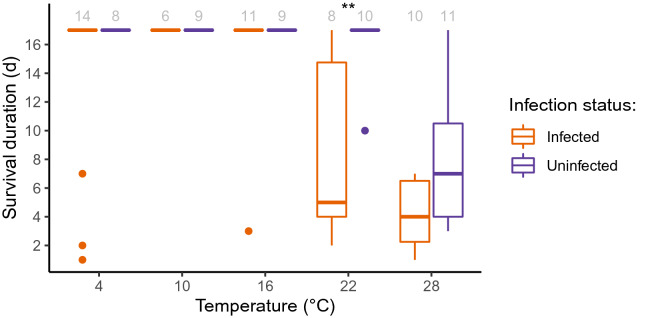
Survival duration of *Littorina littorea* uninfected and infected by *Himasthla elongata* after a 17-day post-acclimation exposure to different temperatures. Asterisks represent significant differences between infected and uninfected individuals (Mann–Whitney-U-Test, Holm-corrected p < 0.01). Gray numbers represent sample sizes.

From the initial 125 snails, 82% survived the acclimation period. Specifically, 94% of the uninfected snails survived as compared to 76% of the infected snails. This left us with a total of 102 snails for Experiment 1, from which 54% were infected by trematodes. Among the infected snails, 11% were infected with *Renicola roscovita* and 89% with *H. elongata,* and no co-infections were detected. Statistical analyses were performed only for snails infected with *H. elongata* since the number of specimens infected by *R. roscovita* was too low to conduct meaningful comparisons. At the beginning of the experiment, 96% of the infections with *H. elongata* were patent (i.e., emergence of cercariae detected), which changed to 74% at the end of the experiment. At the end of the experiment, the percentage of patent infections was lowest at 4 °C (18% of the snails) followed by 10 °C, where 80% of the infected snails presented patent infections. Above 16 °C, patency was higher than 88%. Once acclimatized, *L. littorea* generally died faster at 22 and 28 °C than at colder temperatures (Fig. 2). Snails infected by *H. elongata* survived less than uninfected ones, mainly at 22 °C, where significant differences were detected (Mann–Whitney-U-Test, p < 0.01). At 28 °C, mortality was high in both infected and non-infected snails. However, survival of uninfected individuals was slightly higher (survival range: 3–17 days; Fig. 2), although not significant due to high variability.

### Experiment 1b: Emergence of *Himasthla elongata* cercariae

**Figure 3:**
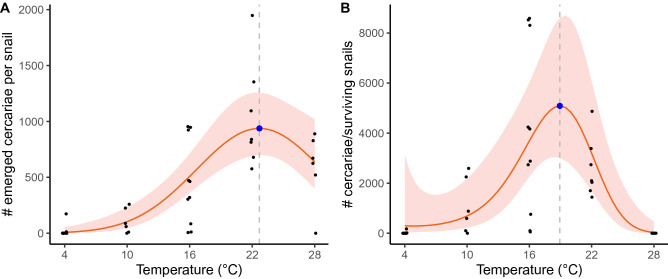
Cercarial emergence per snail (**A**) and net cercarial emergence per snails that survived from an initial population of 10 snails (**B**) over a 3-day incubation experiment in different temperatures. Regressions are based on generalized linear models with distributions of overdispersion-corrected Poisson (**A**) and zero-inflated negative binomial (**B**). The blue dot represents the optimal temperature [22.7 °C (**A**) and 18.9 °C (**B**)] for cercarial emergence [938 cercariae per snail (**A**) and 5088 cercariae per survived snails (**B**)].

The emergence of cercariae was significantly affected by temperature (2nd-degree polynomial: p < 0.001, t = − 5.34; df = 38, R^2^ = 0.85; Fig. 3A, see Supplementary Table S1). The optimal temperature for cercarial emergence was 22.7 °C with an estimated mean of 938 (702–1257) cercariae per snail or 313 (234–419) cercariae per snail per day, which decreased on average by 30% at 28 °C in comparison to 22 °C (Fig. 3A). Almost no emergence was detected at 4 °C. A significant positive linear relationship in response was detected between emerged cercarial encystment and temperature with the highest encystment rate detected at 28 °C (38%) and zero values below 16 °C (p < 0.001; Z = 6.78, R^2^ = 0.78, DF = 38; see Supplementary Table S1 and Supplementary Fig. S1). When correcting cercarial emergence for the survival of snails in each temperature, a negative shift in the optimal temperature by almost 4 °C was observed (Fig. 3B).

### Experiment 2: Self-propelling capacity, survival, and encystment of *Himasthla elongata* cercariae

**Figure 4:**
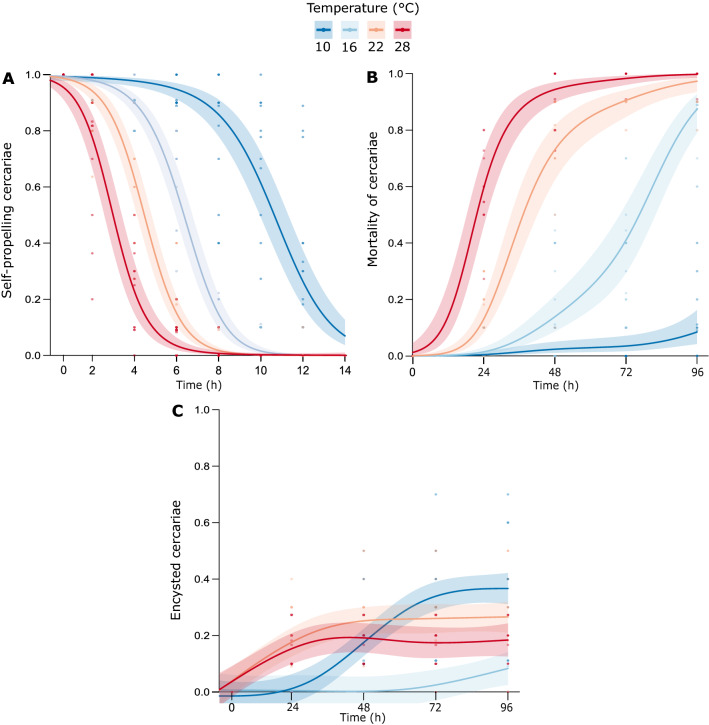
Generalized additive mixed models of *Himasthla elongata* cercariae activity (**A**), mortality (**B**), and encystment (**C**) with temperature (°C) and time (h) as smooth terms. Models explain 87%, 85%, and 78% of the response variance, respectively.

For all the response variables, the nonlinear smooth terms for temperature and time, as well as the tensor product interaction of time and temperature, were significant (p < 0.0001, GAMM *t-*statistic; see Supplementary Table S2). For mortality the time and temperature tensor product interaction was significant to a lesser degree (p < 0.01, GAMM *t-*statistic; Fig. 4B; see Supplementary Table S2). Regarding cercarial self-propelling capacity, a decrease in a sigmoidal manner was observed across time, while increasing temperatures accelerated this decrease (Fig. 4A). The whole model explained 87% of the variance in activity (R^2^ = 0.872; see Supplementary Table S2). For 28 °C the calculated ET_50_ was of 2.96 h (2.56–3.35 h), for 22 °C it was 4.46 h (3.98–4.77 h), for 16 °C 6.31 h (5.95–6.70 h), and for 10 °C 10.65 h (9.98–11.28 h). Moreover, at both 28 °C and 22 °C, the self-propelling capacity ceased completely after 8 h, while at 16 °C and 10 °C cercariae ceased to be active after 10 h and 14 h, respectively. In terms of mortality, an increasing trend was observed across time, and temperature with 85% of the variance explained by the GAMM (Fig. 4B).

Similar to cercarial self-propelling capacity, the increase in mortality along time was enhanced by temperature. Specifically, the estimated half-life of cercariae (LT_50_) was 21.88 h (18.28–25.33 h) at 28 °C, 37.57 h (33.82–42.03 h) at 22 °C, and 76.71 h (70.67–81.90 h) at 16 °C. At 10 °C, maximum mortality of 9% was observed at 96 h with cercariae at this temperature surviving up to 120 h. Finally, pre-mortem encystment of cercariae followed a more complicated pattern. At the warmest temperatures (22 and 28 °C), cercariae started encysting earlier but reached an estimated mean encystment rate of 27% (22–31%) and 18% (13–23%), respectively (Fig. 4C). Meanwhile, cercariae at 16 °C started to encyst later on but reached a higher encystment proportion of 37% (32–42%). At 10 °C, almost no encystment was observed with an estimated mean of 8% (3–14%). Moreover, the optimal temperature for cercariae pre-mortem encystment decreased over time (see Supplementary Fig. S2). In the case of pre-mortem encystment, the model explained 78% of the variance.

### Experiment 3: Infection success

**Figure 5:**
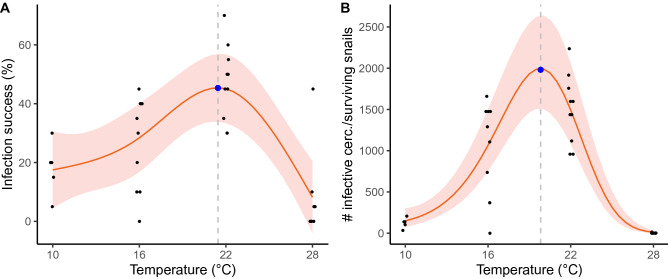
General additive mixed model of *Himasthla elongata* cercarial acute infection success (**A**) and generalized linear mixed model of cercarial net infectivity (**B**) after 24 h of exposure to *Mytilus edulis* s.l. under different temperatures with gaussian (**A**) and zero-inflated negative binomial (**B**) distributions. The blue dot represents the optimal temperature [21.5 °C (**A**) and 19.8 °C (**B**)] for cercariae infectivity [45% infectivity (**A**) and 1933 infective cercariae from surviving snails (**B**)].

Both gross and net infectivity significantly correlated with temperature in a bell-shaped curve (Fig. 5A,B). For acute infection success, the nonlinear smooth term of temperature was significant (p < 0.0001, see Supplementary Table S3). The whole model explained 47% of the variance (R^2^ = 0.469; see Supplementary Table S3). The estimated optimal temperature for infection success was 21.5 °C, with an estimated mean of 45 (30–57)% (Fig. 5A). In terms of organ partitioning, cercariae encysted mostly in the mussel's foot at 10, 16, and 22 °C. Encystment in the mantle was recorded in all temperatures, but it was highest at 22 °C. Encystment in other organs (e.g., adductor and retractor muscles, and intestine) was highest at 22 °C and minimal in the other temperatures. Regarding the gills, no change in encystment was observed among temperatures except for 28 °C where encystment in the gills was absent. At this temperature (28 °C), infection success was the lowest, and only the foot, the mantle, and muscles were infected without any clear difference among these organs. When considering cercarial emergence and the effect that temperature-specific gastropod survival has on cercarial emergence, the optimal temperature for net infective cercariae was reduced by 1.7 °C, with an estimated number of infective cercariae from surviving snails of 1933 (Fig. 5B). For this model, all terms (i.e., first-, second- and third-degree terms) were significant (p < 0.0001, GLMM *z-*statistic; see Supplementary Table S3).

### Summary: log response ratios

**Figure 6:**
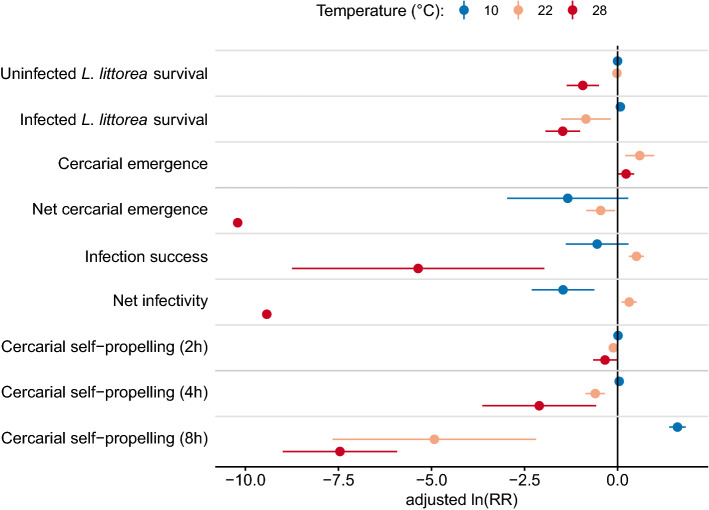
Logarithmic response ratios for crucial traits of the *Himasthla elongata* life cycle in response to temperature deviations. Ratios were calculated and adjusted to small sample sizes according to Lajeunesse^35^ in relation to the baseline temperature of 16 °C. The means of the control temperature for each trait were estimated from the models described in the methods section. Values are given as means and confidence intervals (α = 0.05).

When comparing the treatments to the baseline temperature of 16 °C we can see a differential response of *H. elongata* life cycle stages to temperature (Fig. 6). Specifically, the treatment of 22 °C was beneficial to cercarial gross emergence, infection success, and net infectivity. In contrast, this treatment decreased survival of infected gastropods, net cercarial emergence, and cercarial self-propelling capacity from 4 h onwards. The 28 °C-treatment was detrimental for all traits except for cercarial emergence and activity at 2 h, where the effect was almost neutral (Fig. 6). At 10 °C most traits were unaffected except for net infectivity, which was reduced, and cercarial self-propelling at 8 h, which was greatly enhanced. At 10 °C, no log-response ratio for cercarial emergence was reported since the Geary index suggested by Lajeunesse^35^ was below three. For the self-propelling capacity of cercariae, only the first eight hours are shown since, after this time, the larvae usually lose their self-propelling capacity.

## Discussion

The present study illustrates that the first intermediate host (i.e., gastropod) and cercariae represent a fragile link in the life cycle of trematodes under current extremely warm events and projected end-of-century mean temperatures for temperate systems during summer^36^. The ubiquity of trematodes in the environment and their capacity of modulating complex ecological systems makes their consideration in global warming effects predictions an urgent task. The studied *H. elongata* host–parasite system is a good example of the complex dynamics between closely interacting species such as the trematodes and ecologically relevant benthic species (e.g., the common periwinkle, *L. littorea*, and the blue mussel, *M. edulis* s.l.) under the influence of thermal stress. Our results conclusively showed that the optimal temperature range of parasite performance might be overestimated when looking at individual life cycle components. The infected gastropod's thermal sensitivity and reduced functional lifespan and survival of the cercariae resulted in a decreased overall performance of the parasite with temperatures above the thermal optimum of the host snail.

The trematode-induced gastropod thermal sensitivity is of significant ecological relevance under current and projected thermal regimes for temperate ecosystems as such gastropods play a major ecological role as grazers in their ecosystem. The infected gastropods show substantial mortality already after a few days (i.e., 1–7 days) of exposure to temperatures of 22 °C, projected as mean summer values for the region for the end-of-century^32^, as opposed to uninfected individuals which had no mortality at this temperature, and only started to die after three days exposure to 28 °C. Current summer heatwaves in shallow water Baltic Sea habitats can reach up to 22 °C or even higher for several days^32,34^. This means that even the projected average summer temperatures will be stressful for infected gastropods. Although littorinids are assumed to be resistant to harsh conditions due to their evolutionary history in extreme environments^37–39^, exposure to other stressors (e.g., pathogens) could be fatal to them.

Trematodes pose significant stress to their gastropod host in many ways. First, they are known to castrate their host by chemical interference of the host’s endocrine system and physical destruction in mature infections^31^. Also, cercarial emergence can severely damage the host tissue since they migrate through the skin of the gastropod^31^. Enhanced gastropod mortality by parasites has already been observed for other trematodes. McDaniel^40^ showed that *L. littorea* infected with the trematode *Cryptocotyle lingua* have a reduced heat tolerance. In warm temperatures, infected snails show more mature and heavier infections, while at cold temperatures, infection intensity is reduced, and relatively small rediae can be observed (personal observation). Since higher temperatures can accelerate the development of infections^41^, snails might benefit from colder temperatures where infection development is arrested and poses less stress to the host.

The parasite-enhanced mortality and decreased overall fitness have been observed both in the marine environment and freshwater ecosystems^42–44^. Paull and Johnson^42^ showed that the freshwater trematode *Ribeiroia ondatrae* increased its pathology with warmer temperatures by reducing fecundity in the gastropod *Planorbella trivolvis* (previously *Helisoma trivolvis*). In the same host–parasite system, Paull et al.^43^ showed that infection by *R. ondatrae* increased snail mortality both before and after temperature shifts. These effects of trematodes on their gastropod host can go beyond the individual level and can result in a cascade of effects that compromises the structure and functioning of communities^45^. In the case of *H. elongata*, the prevalence of infection has been reported up to 40% in the southwestern Baltic Sea^46^. A high prevalence of infection combined with decreased thermal tolerance could translate into two possible (mutually non-exclusive) scenarios. First, in the near future, infection plus high temperatures could provoke a significant decrease in gastropod populations and, therefore, the scarcity of a functionally important organism on rocky shores. In the long run—bearing in mind the castrating behavior of *H. elongata*—it could provoke an evolutionary advantage for gastropod populations since thermal stress would select for uninfected (i.e., non-castrated) thermal tolerant individuals which can contribute to the reproduction and persistence of the species.

Acute infection success was also optimal at 22 °C. Higher infectivity with higher temperature has been reported for many cases in the literature for both laboratory and field experiments^20,47–49^. Increasing temperatures accelerate the metabolism of cercariae, providing more ATP for penetrating the host tissue and establishing as metacercariae^15,48^. The infectivity of trematode larvae can also be compromised by a reduction in the filtering activity of the mussel^50^. *Himasthla elongata* targets primarily the foot and the mantle of the mussel, which are usually exposed when the mussel is open. In our case, after offering the cercariae to the mussel, most of them were open with their mantle and foot exposed, except for one mussel at 16 °C and one at 28 °C. Mussel filtration rate has been demonstrated to remain stable up to 24 °C, and beyond this temperature, substantial metabolic depression has been observed^51^. Our data sustain this fact since the infection of the gills remained relatively constant between 10 and 22 °C. Therefore, in our experiment, infectivity might be affected by reduced mussel filtration only at temperature 28 °C.

Reduced activity and survival with increased temperature and time were not a surprise. Previous literature has shown that activity and survival of continuously swimming cercariae are limited to a few hours due to their lecithotrophic character^52^. The inability to feed makes glycogen (the primary energy reserve for cercariae) a limiting factor. Therefore, higher metabolic rates enhanced by warmer temperatures will deplete the energy reserve faster, which manifests into faster loss of activity and, ultimately, death^18^. Accelerated death can be observed when comparing the half-life (ET_50_) of cercariae, which at 28 °C was almost four times lower than at 16 °C. In terms of self-propelling capacity, the ET_50_ values were three times lower at 28 °C than at 16 °C. Self-propelling capacity resulting in an efficient displacement in the water column is an important trait for trematode overall performance. Since himasthlids do not use chemotaxis and do not search actively for a host, they rely on other important factors aiding transmission^50,53^. Such factors include displacement in the water column via self-propelling, phototactic behavior, positive geotropism, which naturally attracts them to benthic invertebrates (i.e., bivalves), and the siphon current from the host itself^29,50^. In this experiment, the capacity for self-propelling generally lasted longer than expected, especially at lower temperatures (10 °C), where cercariae were self-propelling for up to 14 h, although they were displacing themselves very slowly (personal observation). Therefore, conducting behavioral studies that characterize cercariae movement patterns (e.g., swimming velocity and distance moved) in response to temperature might be complementary to self-propelling capacity as indicators of infectivity^54^.

In Experiment 1b, we initially observed increases in the proportion (maximum 40%) of cercariae spontaneously encysted with increasing temperatures. For the marine trematode species *H. elongata,* this type of encystment occurs typically in less than 1% of the cercariae^55,56^, and the factors inducing this type of encystment have not been elucidated yet. Only two studies reported higher in vitro spontaneous encystment in the presence of hemolymph and plasma extracted from mussels^55,57^. However, these factors are only relevant when the cercariae are inside the mussel. In our case, after closely evaluating encystment and mortality rates as a response to time and temperature (i.e., Experiment 2), we can see that cercariae were not only encysting faster but were also dying faster. Therefore, encystment without the presence of the second intermediate host (i.e., spontaneous encystment) seems to be a before-death response. We thus suggest the term *pre-mortem encystment* as a more accurate term to describe this aspect of the life cycle.

Pre-mortem encystment could be useful for the parasite when the second intermediate host is not present. Instead of merely dying, the cercariae transforms into metacercariae, extending the larvae's lifetime for up to 48 h (personal observation). However, reaching the final host in this way is very unlikely. This reduced probability is attributed to the fact that shorebirds get infected more likely when feeding on infected bivalves than when accidentally ingesting metacercariae directly from seawater^58^. Moreover, this external cyst has a thicker impermeable layer that protects against external stressors^15^. A thicker layer does not offer the same advantages as the cyst formed in the bivalve tissue, which has a thinner permeable layer that allows for obtaining nutrients from the host^15^. Another interesting observation was that, even though the cercariae were encysting faster at higher temperatures, the maximum encystment proportion was observed at 16 °C. At higher temperatures, pre-mortem encystment might be hindered by enhanced metabolism leading to a faster loss of energy reserves^59^. The formation of the multiple layers of the cyst requires energy-costly metabolic processes^15^. Similar results were found by Fried and Ponder^60^, who evaluated in vitro encystment of the freshwater echinostome *Echinostoma caproni* at 12, 23, 28, and 37.5 °C. The authors of this study found that maximum encystment in artificial media (Locke’s medium mixed with artificial pond water in a 1:1 ratio) was reached at 23 °C with 78.2% of the cercariae encysted, while at 28 °C it decreased to 43.8% and to 0% at the maximum temperature.

When looking at life cycle components individually, we could hypothesize that trematode transmission might be facilitated in current summer heatwave events and end-of-century temperature scenarios (22 °C for the Baltic during the summer). Cercarial emergence and infectivity were optimal near this temperature (22.7 °C for the emergence and 21.5 °C for infectivity). Nevertheless, this assumption might not hold true for three reasons. First, even though we see a peak of emergence and infectivity, the optimal temperature range is approximately 3–6 °C wide (i.e., optimum between ca. 19–25 °C). The stability of cercariae over a wide thermal range at its optimum has been recently explored and challenges the previous assumption that temperature is the most determinant abiotic factor in the transmission of parasites^48,61,62^. Nevertheless, since the amount of data gathered in this experiment does not allow for an accurate estimation of the optimal temperature range via means of bootstrapping or posterior inference, we highlight the importance of testing the same range of temperatures with higher resolution (i.e., more temperatures). Second, after adjusting cercarial emergence and infectivity to the parasite- and temperature-induced mortality of the gastropod host, we can see that the optimal temperature for emergence and infectivity is shifted to lower temperatures resulting in a costly tradeoff. Specifically, trading first intermediate host survival for higher cercarial emergence as a response to global warming (i.e., prolonged exposure to 22 °C) translates into approximately 41% of loss in net cercarial emergence and 25% of loss in net infectivity in comparison to optimal conditions (i.e., 19 °C). Therefore, this disparity between the thermal performance of the host and the parasite is unstable ground and could translate into a collapse of the host–parasite system overall, as evidenced in other host–parasite systems^10^. The third reason why trematodes will not necessarily benefit from warmer temperatures is the energetic cost that warming implies for the cercariae. Increasing temperature accelerates the loss of self-propelling capacity and death of cercariae, thus tightening the time window at which cercariae are infective. At 22 °C—temperature representing current summer heatwave events and end-of-century projected averages for the Baltic—the half-functional lifespan of cercariae is only 4 h. With mussel beds declining in biomass due to climate change and displacement by invasive species^63,64^, a narrow infective time window represents an obstacle for transmission since a decline in blue mussel populations reduces the probability of cercariae reaching the bivalve host in time before losing infection potential and their chance to continue the life cycle.

Although the overall prognosis for trematode infections in a warming sea does not seem to be auspicious, other scenarios might hold probable. For example, since temperatures are expected to increase gradually until the projected end-of-century scenario is reached, summer thermal averages might initially benefit the parasite in upcoming years. In other words, before we reach a summer thermal average of 22 °C, colder thermal averages (i.e., 18–21 °C) might benefit the parasite initially, and only after, net adverse effects should be expected. Moreover, global warming might create appropriate conditions in seasons that (currently) might be too cold for parasites to proliferate (i.e., in winter). Therefore, we might expect a shift in the season of high infection development and activity instead of an apocalyptic scenario overall. In addition to this, parasites and hosts might be subject to gene adaption and phenotypic plasticity: temperature resistant genotypes and phenotypes of hosts and parasites might be selected in synchrony through adaptive evolution and thus aid in the sustainability of the host–parasite system^65^. Following this line of thought, future research should consider the possibility and the differences in the adaptation capacity of hosts and parasites considering the difference in generation times and the intraspecific genetic variation^42,66^.

Even though we did not evaluate the final and second intermediate host's performance in this study, trematodes show low specificity towards them and thus may find alternative ways to prosper. A low specificity gives an advantage to the trematode by having different options to complete the life cycle. Taking *H. elongata* (Mehlis, 1831) as an example, it has several birds as final hosts, such as *Larus* spp., *Haematopus ostralegus*, and *Somateria mollisima*^29,50^. Regarding the infective larval stage released from the final host (i.e., miracidia), no studies are available specifically on *H. elongata*. However, studies conducted using *H. militaris* showed that, although the half-life of miracidia was significantly reduced at 25 and 30 °C compared to 14 °C, the infectivity of the larvae increased and remained constant at the warmer temperatures^67^. In the case of miracidial eclosion, its proportion increased, and the process was accelerated with increasing temperature (20 and 30 °C) while it was nil at 12 °C^68^, altogether suggesting a high tolerance of miracidia to high temperatures. Moreover, regarding the thermal tolerance of the final host, endotherms have the advantage of modulating their body temperature and are highly mobile, thus being capable of seeking shelter in extreme temperature conditions.

In the second intermediate host’s case, cercariae usually parasitize *M. edulis* s.l., but can also infect other bivalves such as the edible cockle *Cerastoderma edule*^29,69,70^*.* In addition, trematodes encyst as metacercariae—semi-dormant stages of the parasite—in the second-intermediate host^15^. Although metacercariae are known to negatively affect the host’s metabolism, condition index, and biochemistry^71–76^, these larval stages are assumed to pose less stress to the host than rediae, which actively feed on the tissue of the gastropod host^15^. In terms of thermal tolerance, mussels are highly sensitive to thermal exposure and show significant mortality after recurrent heatwave events^77^. Furthermore, since mussels are semi-sessile, they are usually constrained to their habitat and cannot seek shelter as easily as birds or gastropods. However, for infected specimens, recent findings suggest that *H. elongata* metacercarial infections can even protect its host from heat (35 °C compared to 15 °C) at high infection intensities (> 250 metacercariae mussel^−1^)^28^. Although the mechanisms behind this heat-protection are still to be resolved, Selbach et al.^28^ speculate that trematodes might protect the host by pre-equipping it with heat shock proteins.

In summary, by combining multiple traits, we show that the optimal temperature range of parasite performance might be overestimated when looking at individual life cycle components. The thermal sensitivity of the infected gastropod, along with reduced functional lifespan and survival of the cercariae, resulted in a decreased overall performance of the parasite at high temperatures. In addition to this, this study also evaluated the capacity of *H. elongata* cercariae to encyst in the non-host environment as a function of time and temperature for the first time to our knowledge. We determined that increasing temperatures induced faster spontaneous encystment as a consequence of increased cercariae mortality. We show that, as time progresses, the optimal temperature for spontaneous encystment shifts towards colder temperatures down to 16 °C, highlighting the importance of time scale in the life cycle of trematodes.

As with every laboratory study, controlled experiments unavoidably neglect other factors important for the thermal tolerance of marine ectotherms and parasite transmission. Predicting the future of trematodes in a warming sea is difficult due to the numerous factors that are related to warming, which can significantly alter host–parasite dynamics. For example, in warming shallow waters, biotic productivity might be greatly increased^78^. Increased biotic productivity can create opportune conditions that invite birds to aggregate, forage, and increase their infection chances^79–82^. This increase in chances of infection will benefit allogenic parasites such as *H. elongata*, which use birds as final hosts^79,80^. On top of this, the thermal sensitivity of infected snails can be buffered by daily thermal fluctuations, which can provide relief to intertidal organisms, especially at extreme temperatures^83^. Moreover, given that temperature can vary within small spatial scales, snails can mobilize to near macro- and micro-habitats with benign temperatures^84^. On the contrary, our experiments restricted snails to constant temperature conditions. Therefore, there is a possibility of an overestimation of the thermal sensitivity of the gastropod. Additionally, parasites can also influence microhabitat selection, especially by trematodes that influence their host's behavior^85–87^. Some trematode species (e.g., *Maritrema* spp.) can modify the host's behavior so that the host settles in temperatures matching the thermal optimum of the parasite in natural thermal gradients^85^. Therefore, we encourage and highlight the importance of conducting more near-natural experiments on larger scales such as mesocosms and field studies, considering other important factors affecting parasite–host interactions such as salinity, predation, and dilution of free-larval stages^16,50,61,88^.

## Methods

### Collection of hosts

The first intermediate host, the gastropod *L. littorea,* was collected haphazardly by hand from the intertidal at Årøsund, Denmark (55° 15′ 49.0″ N 9° 42′ 24.5″ E) on June 16, 2019. Snails were transported immediately in portable coolers to the laboratory and kept in a climate chamber at 16 °C under a flow-through system of filtered seawater pumped from the Kiel Fjord. Snails were screened for trematode infections by placing one individual per well in 6-well plates filled with 8 mL of filtered seawater. All wells were covered with transparent lids and placed under lamps for 4 h to induce emergence of the parasite. Infected and potentially uninfected snails were kept in separate tanks at 16 °C until the start of the experiment. Snails were fed ad libitum with the seaweed *Fucus vesiculosus* collected from the Kiel Fjord. The second intermediate host, the blue mussel *M. edulis* s.l., was collected from the ‘Kieler Meeresfarm’ aquaculture facility in Kiel, Germany (54° 22′ 59.1″ N 10° 09′ 41.8″ E) on May 28, 2020, where no trematode infections have been found after numerous assessments. Mussels were measured, and individuals measuring 30–40 mm were kept in an 8 L plastic tank filled with filtered seawater at 16 °C. Before starting the experiment, mussels were fed once with 125 ng/L of chlorella powder (*Chlorella vulgaris*, Algomed^®^). To ensure that mussels were uninfected, a sub-sample of 50 mussels was dissected and inspected for metacercarial cysts under a stereo-microscope (Nikon, SMZ1000 body, C-PS160 stand). For a detailed timeline on the collection of hosts and execution of experiments, see Fig. 1.

### Experiment 1a: Host survival

Snails were acclimated between June 26 and July 1, 2019, to 4, 10, 16, 22, and 28 °C, respectively, in 5 different thermo-baths by increasing or decreasing temperature by increments/reductions of 2 °C every 24 h. This range of temperatures was chosen to cover the range of daily variation in temperatures during summer in shallow waters from the Baltic Sea and the expected critical minimum, maximum and optimal temperatures for the parasite^20,34^. Thieltges and Rick^20^ identified 10–25 °C as the relevant temperature range for the Baltic trematode species *Renicola roscovita*. However, since the present study used another trematode species from the Baltic, this range was expanded down to 4 °C and up to 28 °C to ensure coverage of the *H. elongata* tolerance range, should significant differences in the thermal tolerance between these trematode species exist. During the acclimation phase, 10 uninfected and 15 infected adult snails per temperature level of similar shell length (Infected: 2.03–2.56 cm; Uninfected: 2.19–2.80 cm) were distributed in two 1-L tanks per infection group and fed ad libitum with *F. vesiculosus* (Fig. 1). More infected snails were used due to the expected trematode-induced mortality under stressful temperatures. Water was constantly aerated and changed three times a week with temperature equilibrated and previously filtered aerated seawater pumped from the Kiel Fjord. After the acclimation period, snails were transferred individually to 50 mL PLEXIGLAS^®^ beakers with 40 mL of previously aerated and acclimatized filtered seawater pumped from the Kiel Fjord. The photoperiod was set to start of sunrise at 4:00, reaching the maximum experimental intensity of light at 7:00, and sunset starting at 19:00, reaching total darkness at 22:00. Since cercariae are photo-sensitive^89^, we decided to mimic the photoperiod of the season with the highest light intensity in order to avoid underestimations of behavioural responses (i.e., emergence, activity, and infectivity success). Each beaker was covered with a transparent plastic mesh to prevent the snails from escaping. Survival was recorded daily for a total of 17 days. In the case of death, the snail was dissected in order to confirm infection status.

### Experiment 1b: Cercarial emergence

In order to evaluate the dependency of cercarial emergence to temperature, water from the Experiment 1a-snails was changed with clean, temperature equilibrated, and previously aerated filtered seawater pumped from the Kiel Fjord. Snails were incubated in recurrent periods of 12 h during July 2–5, 2019, under artificial light as described for Experiment 1a. After incubation, each beaker (including the ones with uninfected snails) was screened for parasites under the stereo-microscope. Water from the snails that did not shed cercariae was directly replaced with new water. When parasites were spotted, the snail was taken out, and the water was poured into a falcon tube. Afterwards, the beaker was rinsed with 10 mL of 70% ethanol and poured again into the falcon tube to fix the cercariae for later counting. The beaker was rinsed once with tap water and once with filtered seawater, filled with 40 mL of new water and placed back with the snails in the thermo-bath for a new incubation period of 12 h. Cercariae were counted under a stereo-microscope (Nikon, SMZ1000 body, C-PS160 stand) by pouring the content of the falcon tube into a petri dish. Metacercariae were also quantified upon appearance. Afterwards, the falcon tube was rinsed with tap water and poured into the petri dish to ensure all cercariae were counted. In total, cercariae were collected every 12 h over 3 consecutive days. Oxygen levels, salinity, temperature, and pH were monitored before and after water exchanges. During the incubations, snails were fed with 1 cm^2^ of *F. vesiculosus.*

### Experiment 2: Cercarial activity

Snails collected on May 21, 2020, from Årøsund, Denmark, were acclimated during May 24–29, 2020, by increasing or decreasing 2 °C every 24 h. Once the acclimation to the experimental temperatures finished, cercariae were collected on May 30, 2020, by incubating infected snails in 6-well plates filled with 8 mL of clean temperature equilibrated and aerated seawater at the experimental temperatures (10, 16, 22, and 28 °C). Since almost no cercarial emergence occurred at 4 °C, this temperature was not included in the Experiment 2 and 3. After 1 h of incubation of snails under full light stimulus at the acclimation temperatures, fully active cercariae released by 8–12 snails were pooled together to include potential variation in thermal sensitivity among clones^90^. After collection, cercariae were immediately distributed in 12 PLEXIGLAS^®^ beakers (10 cercariae in each beaker) filled with 40 mL of aerated, temperature equilibrated and filtered seawater (Fig. 1). The beakers were distributed between two thermo-baths per temperature level (6 beakers per thermo-bath). The photoperiod resembled the one described for Experiment 1a. During the first 24 h, the activity of cercariae was recorded every 2 h. After the first 24 h, cercariae were evaluated every 24 h until all cercariae were dead or encysted. Activity traits included self-propelling capacity, premortem encystment, and mortality. “Self-propelling capacity” or functional lifespan was defined as cercariae which were swirling and displacing themselves in the water. “Premortem encystment” is the encystment of cercariae without the presence of the second intermediate host, which was identified by the formation of a defined opalescent cyst inside which the larvae could move^15^. Cercariae were categorized as “dead” when no movement was detected for 15 s after mechanical stimulus with a thin needle. In order to monitor water parameters (salinity, temperature, pH, and dissolved oxygen) without disturbing the cercariae with the multimeter probe (WTW 3630 IDS, Kaiserslautern, Germany), an additional seawater filled beaker per thermo-bath was added.

### Experiment 3: Cercarial infectivity

Infectivity of *H. elongata* was performed as described in Bommarito et al.^61^. In brief, on June 1, 2020, 10 mussels (*M. edulis* s.l.) per temperature measuring 30–40 mm of length were distributed among 50 mL PLEXIGLAS^®^ beakers, each filled with 40 mL of aerated, temperature equilibrated, and filtered seawater (Fig. 1). Each set of ten beakers (with mussels), were distributed between two thermo-baths (5 beakers per thermo-bath) previously set to the experimental temperatures (10, 16, 22 or 28 °C). The same photoperiod described for Experiment 1a was used. Before offering the cercariae to the mussels, 2 mL of a chlorella powder solution (2.50 µg/L; *C. vulgaris*, Algomed^®^) was added to each beaker (final concentration: 100 ng/L) to induce mussel filtering. From the same set of snails acclimatized in Experiment 2, cercariae were collected applying the same approach as described for Experiment 2. Nevertheless, since a significant number of snails died in the 28 °C treatment, the minimal number of snails possible (*n* = 4) was used for cercariae collection in all treatments. After cercariae collection, 20 fully active cercariae were pipetted in each beaker as close as possible to the mussel inhalant siphon. They were then incubated for 24 h to ensure complete cercarial encystment^91^. Mussels were removed from the experimental containers and kept at − 80 °C until infection intensity evaluation. The intensity of infection was evaluated by counting metacercariae in whole soft body squash preparations under a stereo-microscope (Nikon, SMZ1000 body, C-PS160 stand).

### Statistical analyses

All analyses were performed in R (version 4.0.2), RStudio© 1.3.1073 (2009–2020 RStudio, PBC). For Experiment 1a, the significance of differences between the means of infected and uninfected hosts’ survival in each temperature was tested with a Mann–Whitney-U test with Holm-corrected p-values. The variance of gross cercarial emergence, encystment proportion, and net cercarial emergence was modeled in response to temperature as a continuous predictor. For cercarial emergence (Experiment 1b), a generalized linear model was applied with Poisson distribution and a second-degree polynomial term (considering the complex nonlinear effect of temperature) using the “glm” function from the “stats” package. The assumption of residual independence was tested by inspecting response (ordinary residuals, *y*_*i*_* − μ*_*i*_), deviance, Pearson, and scaled-Pearson residuals against predicted values and temperature^92^. However, since over-dispersion was detected, a correction to the standard errors was performed using a quasi-GLM model^92^. For the proportion of encysted cercariae per snail, over-dispersion was also detected. In this case, a negative binomial GLM was chosen over a quasi-GLM with Poisson distribution based on the log-likelihood test and the dispersion parameter^92^. Net cercarial emergence was calculated based on a hypothetical population of 10 snails using Eq. 1.

1 $${N}_{E,x}=n\times {E}_{i,x}\times {p}_{S,x},$$

where ${N}_{E,x}$ is the net cercarial emergence (# cercariae emerged from survived snails at temperature *x*); $n$ is the number of snails in a population (here, assumed to be 10); ${E}_{i,x}$ is the # cercariae emerged per snail (replicate *i*) at temperature *x*; ${p}_{S,x}$ is the snails survival probability at temperature *x*. Although it is known that cercarial emergence rate can change over time^61,89^, we assume that the emergence per individual in this case represent the natural variability in emergence patterns since snails were naturally infected and are, therefore, not synchronized.

The variation in net cercarial emergence was modeled using a generalized linear model with zero-inflated negative binomial distribution with linear parametrization and a third-degree polynomial term with the function “glmmTMB” from the “glmmTMB” package^93^. Model suitability was evaluated using the residual diagnostics tool from the “DHARMa” package^94^, which includes quantile–quantile plots with KS test, outlier and dispersion as added tests, and a residual plot against predicted values with a built-in quantile regression to detect deviations from normality^94^.

Variations in the proportion of active, dead and encysted cercariae (evaluated in Experiment 2) were modeled as functions of time and temperature as continuous variables using general and generalized additive mixed models (GAMM) with restricted maximum likelihood (REML) as fitting method using the “gam” function from the “mgcv” package^95,96^. GAMMs were selected over GLMMs to allow for the needed flexibility in modeling the variance in the response; the response variables varied differently and non-linearly along time in different temperature treatments, specially encystment rates. A binomial distribution with weights on the number of cercariae per beaker was used for modeling cercarial self-propelling and mortality, and a gaussian distribution was used for cercarial encystment. Temperature and time were included as smooth terms and their interaction as a tensor product (i.e., non-isotropic smooth), which allows the modeling of an interaction between variables in different units such as time and temperature^95^. The attributes “thermobath” and “sample id” were included as random (intercept) effects in the global models and time as AR-1 autocorrelation structure to correct for potential dependency in the residuals along time. Model validation was performed by the “gam.check” function from the “mgcv” package and evaluating the residuals histogram plots and boxplots of residuals against each term. All global GAMM models were reduced to having only “sample id” as random factor since the random effects from “thermobath” were not significant. Since no among-residual dependency along time was detected for cercarial self-propelling and mortality (Fig. 4A,B), no autocorrelation structure was included in the models. In the case of cercarial encystment (Fig. 4C), temporal autocorrelation was detected and was therefore corrected for in the model. For cercariae self-propelling capacity the effective time when 50% of the response was reached (ET_50_) was calculated with imageJ based on the plotted model estimates against time. A similar approach was taken to calculate the half-life or lethal time when 50% of the cercariae were dead (LT_50_).

For experiment 3, the variation in infection success as a function of temperature was modelled as a continuous variable using GAMM with REML as fitting method. Temperature was included as a smooth term and thermobath as a random (intercept) effect. GAMM was selected over GLMM because it offered the best compromise between model performance and biological plausibility. Model suitability was evaluated using the residual diagnostics tool from the “DHARMa package”. Net cercarial infectivity was calculated using the Eq. (2).

2 $${N}_{I,x}=\overline{{N }_{E,x}}\times {I}_{i,x},$$

where ${N}_{I,x}$ is the net infectivity (# infective cercariae adjusted to the proportion of survived snails per temperature at temperature *x*); $\overline{{N }_{E,x}}$ is the mean net cercarial emergence at temperature *x* (estimated from the corresponding GLM model); ${I}_{i,x}$ is the infection success (replicate *i*) at temperature *x*.

The variation in net infectivity as a response to temperature was modeled using a generalized linear model with a third-degree polynomial term, thermobath as random factor and zero-inflated negative binomial distribution with linear parametrization using the “glmmTMB” function. Model suitability was evaluated using the residual diagnostics tool from the “DHARMa” package.

For all models (with the exception of zero-inflated models) marginal and conditional R-squares were extracted using the “r.squaredGLMM” function of the “MuMIn” package^97^. For models with a log-link function the trigamma method was used to calculate pseudo-R^2^ and for binomial distribution theoretical pseudo-R^2^ was used^98^. For zero-inflated models, R^2^ were extracted with the function “r2_zeroinflated” from the “performance” package^99^. Optimal temperatures for gross and net cercarial emergence, and gross and net infectivity of cercariae were estimated from the respective models using the function “predict” from the “car” package^100^. The normality of distributions was tested through a Shapiro–Wilk test and further evaluated with histograms and boxplots.

In order to be able to compare among the measured response variables or trematode performance traits at different temperatures, the logarithmic response ratio was calculated in relation to the mean response from the control temperature of 16 °C. The means of the control temperature for each trait were estimated from the above-described models. This was performed according to Lajeunesse^35^, who developed an adjustment to the widely used response ratio described by Hedges et al.^101^. The adjustment procures the avoidance of biases from small sample sizes (n < 15). To this matter, Eq. (3) was applied.

3 $${RR}^{\Delta }=\mathrm{ln}\left(\frac{{X}_{T}}{{\overline{X} }_{C}}\right)+\frac{1}{2}\left[\frac{{\left({SD}_{T}\right)}^{2}}{{N}_{T}{\overline{X} }_{T}^{2}}-\frac{{\left({SD}_{C}\right)}^{2}}{{N}_{C}{\overline{X} }_{C}^{2}}\right],$$

where ${N}_{T/C}$ is the sample size in treatment/control; ${RR}^{\Delta }$ is the adjusted log response ratio; ${SD}_{T/C}$ is the standard deviation of treatment/control sample; ${\overline{X} }_{C/T}$ is the estimated mean response of control/treatment; ${X}_{T}$ is the response of a sample from a treatment.

Next to the log ratio, Lajeunesse^35^ recommends the employment of a small-sample size adjusted Geary index for both the control and treatment groups as diagnostic tool to validate log response ratios. The adjusted Geary index can be checked using the Eq. (4).

4 $$\frac{\overline{X}}{SD}\sqrt{N }\ge 3.$$

## Supplementary Information

Supplementary Information.

## Data Availability

All of the data that support the findings of this study are available from the corresponding author on reasonable request.
