# Supplementary material for: Heat sensitivity of first host and cercariae may restrict parasite transmission in a warming sea
Source: Sci Rep. 2022 Jan 21;12:1174. doi: 10.1038/s41598-022-05139-5 (PMC8782892; doi:10.1038/s41598-022-05139-5)
Supplement: Supplementary file 1 — Supplementary Information. [file 41598_2022_5139_MOESM1_ESM.pdf]

Heat sensitivity of first host and cercariae may restrict parasite transmission in a warming sea

Díaz-Morales, Dakeishla M.[*]; Bommarito, Claudia; Vajedsamiei, Jahangir; Grabner, Daniel S.; Rilov, Gil; Wahl, Martin; Sures, Bernd

SUPPLEMENTARY MATERIAL

Supplementary material

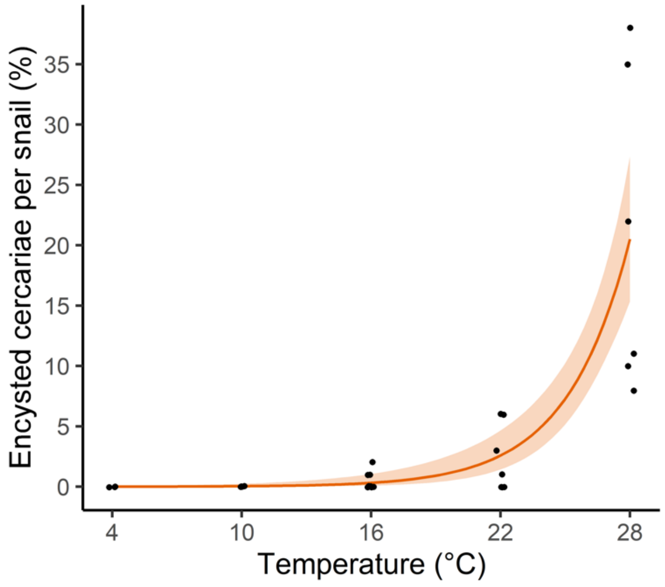

Supplementary Figure S1. Generalized linear model of cercariae encystment after emergence from snail with negative binomial distribution after a 3-day incubation period to different temperatures.

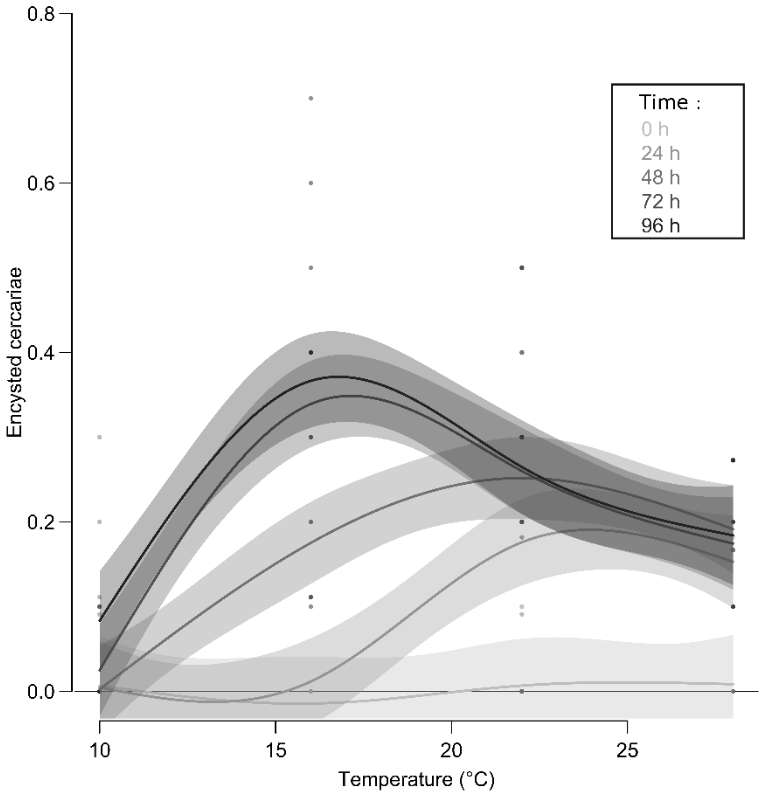

Supplementary Figure S2. Generalized additive mixed model of cercariae per-mortem encystment with time (h) and temperature (°C) as smooth terms.

Supplementary Table S1: Regression parameters estimated from Generalized Linear Models applied on the variance of cercarial emergence, cercariae encystment, and net cercarial emergence.

Response variable	Model/Distribution	DF	Predictor	Estimate	SE	t	z	p-value	R^2
Cercarial emergence	Poisson*	38	Intercept	4.92	0.36	13.77		<0.001	0.852
			Temperature	11.26	2.45	4.59		<0.001	
			$Temperature^2$	-5.34	1.45	-3.69		<0.001	
Cercariae encystment	Negative binomial	27	Intercept	-6.59	1.24		-5.31	<0.001	0.776
			Temperature	0.34	0.05		6.78	<0.001	
Net cercarial emergence	Negative binomial	34	Intercept	6.51	0.48		13.65	<0.001	0.775
			Temperature	-0.7	3.58		-0.20	0.845	
			$Temperature^2$	-9.53	2.45		-3.90	<0.001	
			$Temperature^3$	-4.35	2.02		-2.15	<0.05	
	Zero-inflation		Intercept	5.04	171.49		0.03	>0.05	
			Temperature	-1.22	45.97		-0.03	>0.05	

*corrected for overdispersion; DF= degrees of freedom; SE=standard error

Supplementary Table S2: Approximate significance level of smoothing functions from General(ized) Additive Mixed Models applied on the variance of cercariae activity, mortality, and encystment.

Response variable	Model/Distribution	Predictor	EDF	Chisq	P-value	R^2
Cercariae activity	Binomial	s(Time)	2.92	607.03	<0.001	0.872
		s(Temperature)	2.02	165.90	<0.001	
		ti(Time, Temperature)	2.95	70.74	<0.001	
		s(Sample_id)*	33.63	122.28	<0.001	
Cercariae mortality	Binomial	s(Time)	1.94	192.96	<0.001	0.811
		s(Temperature)	1.68	113.30	<0.001	
		ti(Time, Temperature)	3.40	15.97	<0.01	
		s(Sample_id)*	30.71	100.96	<0.001	
**Response variable**	**Model/Distribution**	**Predictor**	**EDF**	**F**	**P-value**	R^2
Cercariae encystment	Gaussian	s(Time)	2.44	108.15	<0.001	0.778
		s(Temperature)	2.54	12.65	<0.001	
		ti(Time, Temperature)	8.37	19.37	<0.001	
		s(Sample_id)*	37.9	5.52	<0.001	

*random effect; EDF= effective degrees of freedom

Supplementary Table S3: Regression coefficients estimated from Generalized Additive Mixed Model (GAMM) applied on the variance of acute infection success and Generalized Linear Mixed Model (GLMM) on net acute infection success.

**Response variable**	**Model/Dist**	**Predictor**	**Estimate**	**SE**	**t**	**p-value**	**mR^2**	**cR^2**
Acute infection success	GAMM/ Gaussian	Intercept	26.35	2.69	9.80	< 0.0001	0.469	0.469
		**Predictor**	**EDF**	**F-value**	**P-value**			
		s(Temperature)	2.81	10.85	0.0001			
		s(Thermobath)	0.95	0.20	0.2625			
**Response variable**	**Model/Dist**	**Predictor**	**Estimate**	**SE**	**z**	**p-value**	**mR^2**	**cR^2**
Net infection success	GLMM/ Negative binomial	Intercept	5.58	0.18	30.18	< 0.001	0.786	0.793
		Temp	-6.14	1.23	-5.01	< 0.001		
		Temp^2	-9.80	1.06	-9.25	< 0.001		
		Temp^3	-2.47	0.67	-3.70	< 0.001		
	GLMM/ Zero-inflation	Intercept	-2.08	3.61	-0.58	0.565		
		Temp	-0.07	0.22	-0.31	0.756		

Dist= distribution family; Temp= temperature; DF= degrees of freedom; SE= standard error; mgR^2= marginal r-square (variance explained by fixed effects); cR^2= conditional r-square (variance explained by fixed effects and random effects)
